# Déhiscence post-traumatisme sur greffe de cornée à la Clinique Ophtalmologique de l’Hôpital Aristide Le Dantec Dakar

**DOI:** 10.11604/pamj.2020.37.23.22385

**Published:** 2020-09-07

**Authors:** Joseph Matar Mass Ndiaye, Aboubacry Sadikh Sow, Habsa Kane, Jean Pierre Diagne, Aïssatou Magatte Wane, Aly Mbarka Ka, Elhadji Amadou Ba, Paule Aida Ndoye Roth

**Affiliations:** 1 Clinique Ophtalmologique, Hôpital Aristide le Dantec Dakar Sénégal, 30 rue Pasteur, Dakar, Sénégal,; 2 Clinique Ophtalmologique, Centre Hospitalier Abass Ndao, Avenue Cheikh Anta Diop, Dakar, Sénégal,; 3 Service Ophtalmologie, Hôpital d’Enfants de Diamniadio, N2, Sénégal

**Keywords:** Cornée, kératoplastie, déhiscence, traumatisme, Cornea, keratoplasty, dehiscence, trauma

## Abstract

La déhiscence post traumatique constitue une affection rare et qui, souvent, assombri le pronostic visuel des patients greffés. Elle peut survenir après toute kératoplastie transfixiante même pour des traumatismes de faible énergie. Il s’agit de deux patients aux antécédents de kératoplastie, reçus en urgence pour l’œil rouge douloureux après un traumatisme. Leur examen objectivait une baisse de l’acuité visuelle, une désinsertion subtotale du greffon et une hypotonie oculaire majeure. Après un traitement médical symptomatique, ils ont bénéficié d’une réparation chirurgicale respectivement après 16h et 72h. Le pronostic visuel était meilleur pour le patient n°2, qui a retrouvé une acuité visuelle utile. Le patient n°1 n’ayant pas pu améliorer son acuité visuelle du fait de l’importance de l’atteinte du greffon et d’un décollement de rétine associé. Ces deux observations cliniques nous permettent de mettre en exergue le mauvais pronostic des traumatismes oculaires survenant sur une greffe de cornée. Le pronostic est orienté par la survie, la viabilité du greffon et l’existence de lésions associées. La meilleure prévention de cette affection passe par l’éducation, l’information et la communication des patients surtout jeunes.

## Abstract (en)

Post-traumatic dehiscence is a rare condition often compromising the visual outcome of transplanted patients. It can occur after transfixing keratoplasty even after low-energy trauma. We here report two cases of patients with a history of keratoplasty, admitted to the hospital with painful red eye due to trauma. Patients’ assessment objected decrease in visual acuity, subtotal graft displacement and major ocular hypotonia. After symptomatic medical treatment, they underwent surgical repair after 16 hours and 72 hours respectively. Visual prognosis was better in patient 2, who reported proper visual acuity. In patient 1 visual acuity didn’t improve due to the severity of graft damage and the associated retinal detachment. The purpose of this study was to highlight the poor outcome of patients with ocular trauma after corneal graft. Prognosis was determined by survival, feasibility of grafting and the presence of associated lesions. Education, information and communication campaigns to raise awareness of this condition, especially among young patients, is the best way of preventing it.

## Introduction

La greffe de cornée ou kératoplastie est une transplantation chirurgicale d’un fragment de cornée. Elle peut être transfixiante, intéressant toute l’épaisseur cornéenne, lamellaire antérieure ou postérieure avec conservation des couches profondes de la cornée. Les kératoplasties lamellaires antérieures profondes permettent de traiter les lésions jusqu’à la membrane de Descemet, et les kératoplasties lamellaires postérieures permettent de prendre en charge les lésions endothéliales. La rupture traumatique peut survenir même pour des traumatismes de moindre énergie. Cette déhiscence constitue une affection rare et qui, souvent, assombrit le pronostic visuel des patients greffés.

## Patient et observation

Il s’agit de rapporter deux observations de patients reçus aux urgences pour traumatisme oculaire, et qui ont présenté une déhiscence cornéenne.

**Figure 1:**
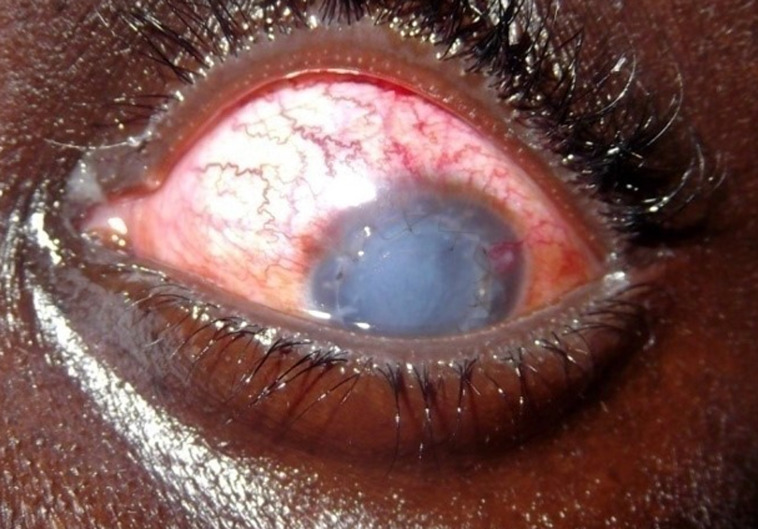
aspect au 7^e^ jour postopératoire du patient n°1

**Figure 2:**
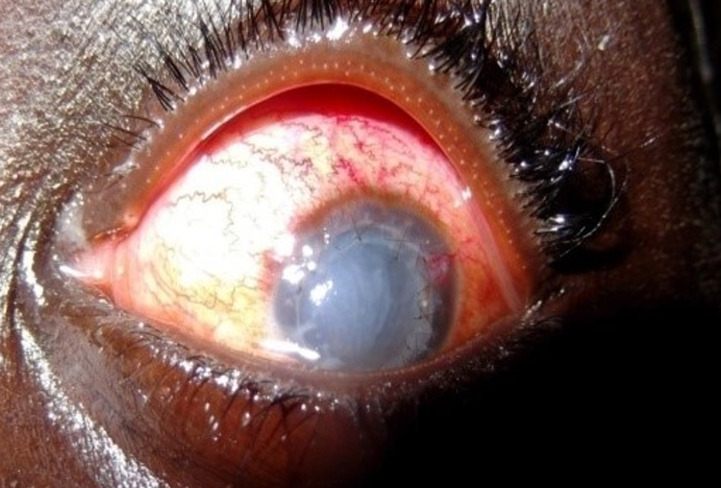
aspect au 30^e^ jour postopératoire du patient n°1

**Observation n°1:** il s’agissait d’un patient de 22 ans, aux antécédents de kératocône traité par kératoplastie transfixiante bilatérale en 2008 à l’œil droit et en 2009 à l’œil gauche en Tunisie. Il avait été reçu en urgence au décours d’un traumatisme survenu lors d’un match de football. L’examen avait trouvé une baisse de l’acuité visuelle limitée à une perception lumineuse, une hyperhémie conjonctivale avec injection ciliaire, une désinsertion subtotale du greffon cornéen avec rebord inféro nasal en place, une issue de vitré. On notait une hypotonie oculaire majeure. Une réparation chirurgicale sous anesthésie locorégionale avait été réalisée dans un délai de 16h après le traumatisme. Elle consistait dans un premier temps en une vitrectomie antérieure, puis en une suture du greffon par six points séparés associés à un surjet au fil nylon 10.0. Une reformation de la chambre antérieure par une bulle d’air a également été faite (Figure 1). Le traitement médical postopératoire avait comporté de la dexaméthasone en collyre en raison d’une goutte par heure et un antibiocorticoïde pommade pour le soir. Par voie orale, une antibiothérapie à base de ciprofloxacine 1,5g par jour et de la bêtaméthasone 4mg par avait été administré. A J7 postopératoire, le greffon était opacifié et l’examen montrait une hypothalamie et une hypotonie ayant empêché la réalisation d’une échographie oculaire mode B. A un mois, l’acuité visuelle demeurait nulle, le greffon était devenu totalement opacifié, et on notait un début de vascularisation cornéenne (Figure 2). L’échographie mode B objectivait un décollement partiel de la rétine. Le patient était retourné par la suite en Tunisie pour une reprise chirurgicale.

**Figure 3:**
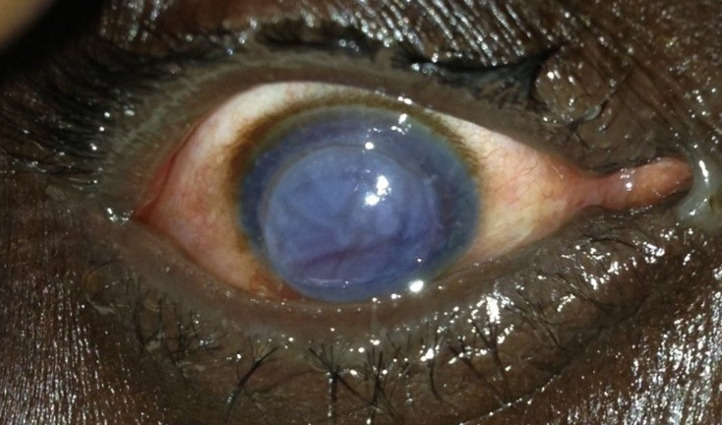
aspect préopératoire du patient n°2

**Figure 4:**
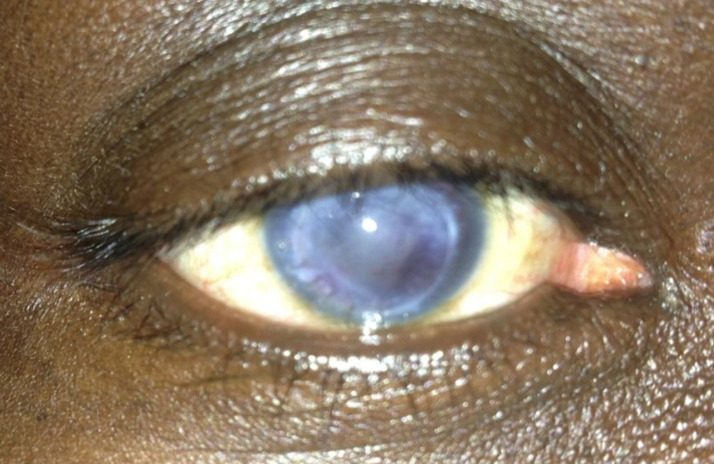
aspect au 30^e^ jour postopératoire du patient n°2

**Observation n°2:** il s’agissait d’un patient de 50 ans, aux antécédents de dégénérescence cornéenne post chirurgie de cataracte à l’œil droit ayant bénéficié d’une kératoplastie transfixiante en 2004 en Italie. Il avait été reçu en urgence pour un traumatisme oculaire de l’œil droit par coup de poing. Son examen avait trouvé une acuité visuelle limitée à une perception lumineuse, des sécrétions séro-muqueuses au cantus interne, une tuméfaction palpébrale modérée et une hyperhémie conjonctivale. On notait une désinsertion du greffon cornéen allant de 2h à 7h (Figure 3), une issue du vitré à travers la brèche et une hypotonie oculaire majeure. A l’œil adelphe, l’examen ophtalmologique était sans particularités. Le malade avait été hospitalisé et avait bénéficié d’un traitement médical à base d’antalgiques en intraveineuse, d’antibiotiques (quinolone) et d’anti-inflammatoires stéroïdien (bêtaméthasone) en per os. La réparation chirurgicale était faite, à J3 d’hospitalisation sous anesthésie locorégionale, par 12 points séparés au nylon 10.0. Les suites opératoires étaient simples. A J7 postopératoire le greffon était bien en place avec une opacification modérée. A J30 postopératoire le greffon s’était bien éclaircit avec une acuité visuelle à 1/10 (Figure 4).

## Discussion

Ces deux observations cliniques nous permettent de mettre en exergue le mauvais pronostic d’un traumatisme oculaire survenant sur une greffe de cornée. Le globe oculaire après kératoplastie transfixiante présente, en effet, un haut risque de rupture du greffon avec une perte fonctionnelle. Cette déhiscence pourrait même survenir pour des traumatismes de faible énergie. Elle surviendrait le plus fréquemment au niveau de la jonction hôte-greffon qui représenterait une zone de grande fragilité [1-3]. Cette jonction hôte-greffon ne cicatriserait pas ad integrum, et la cornée n’acquerrait jamais la solidité d’une cornée normale. La résistance et la solidité du greffon dépendent des sutures, puis progressivement de la cicatrisation qui permet d’unir la jonction hôte et greffon [4]. Deux quadrants sont généralement intéressés par la déhiscence [1, 3]. Chez nos deux patients la rupture est relativement étendue intéressant plus de deux quadrants. Les mécanismes les plus fréquents sont un traumatisme par un objet contondant ou une chute avec réception face contre sol [2, 5]. Certaines périodes apparaissent plus à risque, notamment le premier mois postopératoire durant lequel surviennent près de 30% des ruptures, et la première année au cours de laquelle 64% des cas sont retrouvés [4].

Le pronostic est orienté par la survie et la viabilité du greffon. Ceci est favorisé par un geste rapide et adéquat dans le contexte d’urgence. Dans les pays en voie de développement, on pourrait noter un délai d’intervention relativement long du fait de la rareté des centres spécialisés dans les établissements publics de santé. Plusieurs auteurs ont noté une récupération fonctionnelle compromise avec une acuité visuelle à 1/10 dans la majorité des cas. Le pronostic est d’autant plus assombri qu’il existe des lésions associées liées au traumatisme causal. Tel fut le cas pour nos patients qui ont présenté une issu de vitré et un décollement de rétine pour un [1, 3, 6]. La kératoplastie sur kératocône constitue un facteur de risque majeur du fait du jeune âge et donc du niveau d’activité du patient, mais aussi du fait d’une cicatrisation de mauvaise qualité notamment au niveau de la zone de fragilité que représente la jonction hôte-greffon [4].

## Conclusion

La greffe de cornée constitue un facteur de fragilisation du globe. Tout traumatisme, quel qu’en soit le mécanisme, risque de compromettre le bénéfice anatomique et fonctionnel de la kératoplastie. Le manque de centres spécialisés dans la prise en charge des pathologies de la cornée, de même que l’absence de banque d’yeux, aggravent le pronostic de ces traumatismes dans notre contexte. La meilleure prévention passe par l’éducation, l’information et la communication des patients ayant subi une kératoplastie.
